# Supplementary material for: Novel Stenotrophomonas maltophilia Bacteriophage as Potential Therapeutic Agent
Source: Pharmaceutics. 2022 Oct 18;14(10):2216. doi: 10.3390/pharmaceutics14102216 (PMC9612306; doi:10.3390/pharmaceutics14102216)
Supplement: Supplementary file 1 [file pharmaceutics-14-02216-s001.zip › pharmaceutics-1910595-supplementary.pdf]

pharmaceutics

MDPI

Supplementary Materials: Novel *Stenotrophomonas maltophilia* Bacteriophage as Potential Therapeutic Agent

Rima Fanaei Pirlar, Jeroen Wagemans, Fabian Kunisch, Rob Lavigne, Andrej Trampuz and Mercedes Gonzalez Moreno

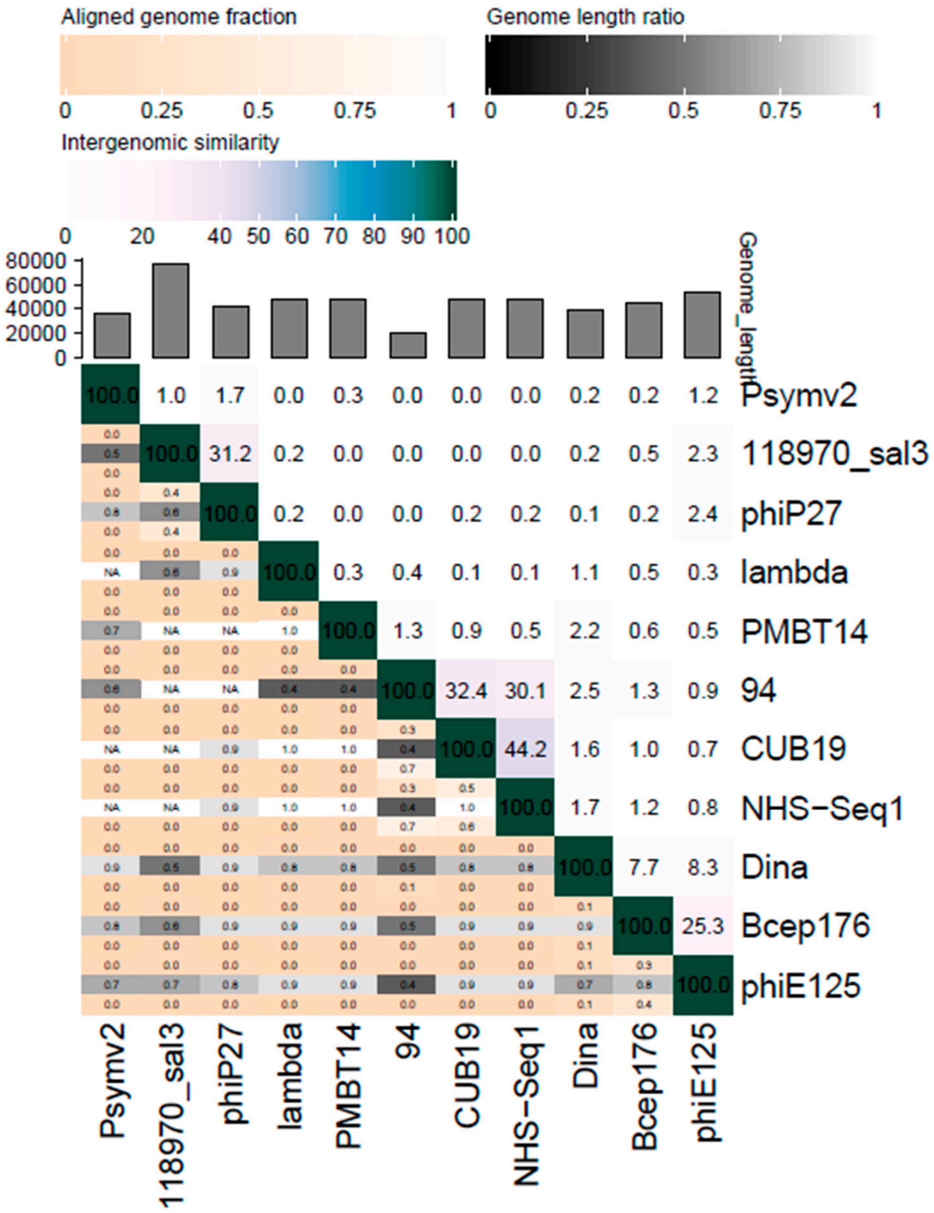

Figure S1. Taxonomic evaluation of CUB19. Heatmap generated with VIRIDIC tool integrating the intergenomic similarity values of CUB19 to related bacteriophages.

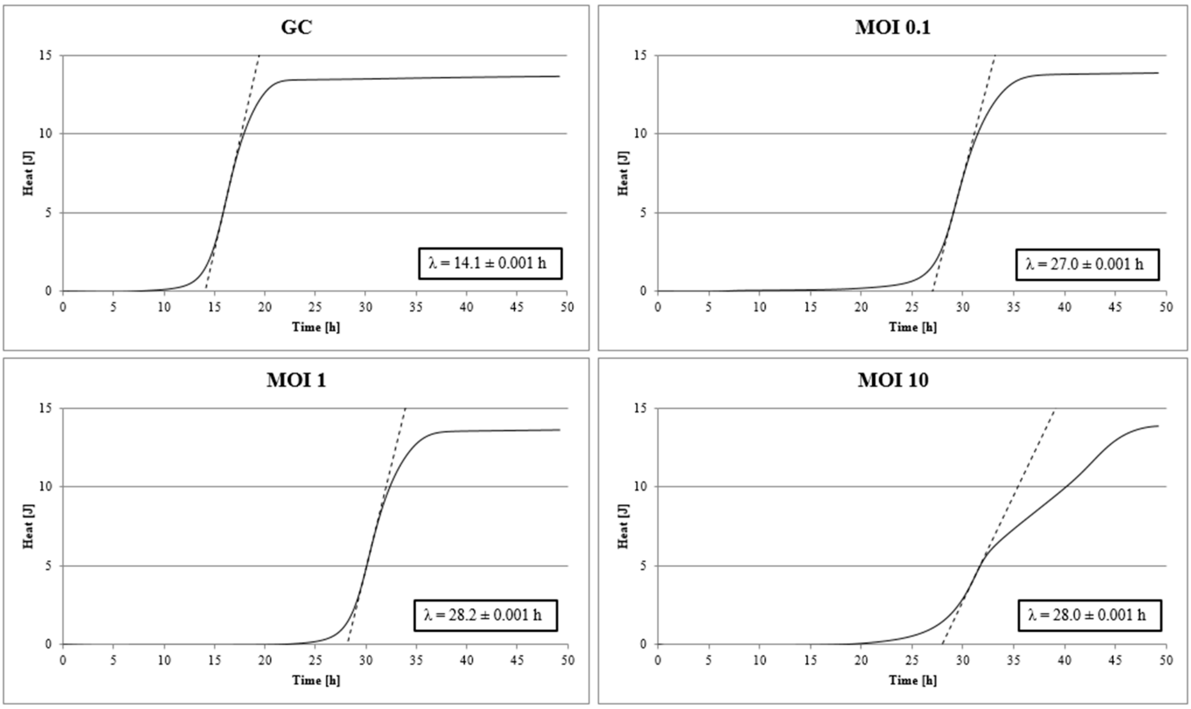

Figure S2. Lag time (λ, h) determination from the heat (J) production of planktonic *S. maltophilia* STM-19 (10^5 CFU/ml) co-incubated with phage CUB19 at different MOI (GC, growth control, not exposed to phage). λ refers to the duration of the lag phase measured as the time from the start of the experiment to the interception of a line tangent to the maximum growth rate point and the baseline.
